# Reproductive outcomes of office hysteroscopic metroplasty in women with unexplained infertility with dysmorphic uterus

**DOI:** 10.4274/tjod.30111

**Published:** 2018-09-03

**Authors:** Bülent Haydardedeoğlu, Gülşen Doğan Durdağ, Seda Şimşek, Pınar Çağlar Aytaç, Tayfun Çok, Esra Bulgan Kılıçdağ

**Affiliations:** 1 Başkent University, Adana Dr. Turgut Noyan Practice and Research Hospital, Clinic of Obstetrics and Gynecology, Adana, Turkey

**Keywords:** Dysmorphic uterus, T-shaped uterus, office hysteroscopy, obstetric outcome, unexplained infertility

## Abstract

**Objective:**

The correlation between dysmorphic uterus and infertility still remains enigmatic. We evaluated the reproductive outcomes of metroplasty via office hysteroscopy in unexplained infertile women with dysmorphic uteri.

**Materials and Methods:**

In this retrospective cohort study, metroplasty via office hysteroscopy using a bipolar system was performed to 272 women with unexplained infertility with dysmorphic uteri from January 2013 to January 2016. Of all the patients, 162 had primary infertility, and 110 had secondary infertility.

**Results:**

In the primary infertility group, the clinical pregnancy rate was 45.68% (74/162) and the live birth rate was 38.9% (63/162), and in the secondary infertility group, the clinical pregnancy rate was 55.45% (61/110) and the live birth rate was 49% (54/110) after metroplasty. In the secondary infertility group, the miscarriage rate and especially the ectopic pregnancy rate declined dramatically [from 84.5% (93/110) to 9.8% (6/61) and from 15.5% (17/110) to 1.6% (1/61), respectively] (p<0.01).

**Conclusion:**

Reproductive outcome can be impaired by Müllerian anomalies, hence, infertile women with dysmorphic uteri should undergo hysteroscopy to improve reproductive outcomes. Our study demonstrated that office hysteroscopic metroplasty of a dysmorphic uterus might improve fertility, particularly in patients with unexplained infertility with dysmorphic uteri, which was an ignored factor previously. Office hysteroscopy is an alternative option in terms of non-invasive procedure.

**PRECIS:** The correlation between dysmorphic uterus and infertility still remains enigmatic. We evaluated the reproductive outcomes of office hysteroscopic metroplasty in unexplained infertile women with dysmorphic uterus. Our study demonstrated that office hysteroscopic metroplasty of a dysmorphic uterus might improve fertility, particularly in unexplained infertility patients with dysmorphic uterus.

## Introduction

A dysmorphic uterus, which was formerly known as “T-shaped uterus” in the American Fertility Society classification of anomalies of the Müllerian duct, is denoted as a second-class (Class U1) uterine anomaly in the European Society of Human Reproduction and Embryology (ESHRE) and the European Society for Gynaecological Endoscopy (ESGE) (ESHRE/ESGE) consensus on the classification of congenital genital tract anomalies, and it leads to poor reproductive and obstetric outcomes^(1,2)^. Although the prevalence of dysmorphic uterus is not yet known, the reproductive performance of a dysmorphic uterus after hysteroscopic metroplasty is poorly documented; only five trials have been reported to date^(3,4,5,6,7)^. In the past, operative hysteroscopy for metroplasty was used to expand the uterine cavity; currently, office hysteroscopy with a bipolar system is used, which appears to have minimally invasive effects, thus making it feasible for metroplasty^(7)^. The encouraging results of the case series of 114 women remain in small numbers in the literature^(3,4,5,6,7)^. The correlation between dysmorphic uterus and infertility still remains enigmatic. No study has definitely defined such a uterus as being the primary reason for infertility. Although the poor obstetric outcomes of Müllerian anomalies are well-known issues, their reproductive performance in terms of infertility has been relatively ignored. Nevertheless, the lesser expansion of a unicornuate uterus is seen as the cause for infertility. The relationship between a dysmorphic uterus and infertility is not clarified exactly in the literature due to the absence of evidence on this topic. The aim of our study was to evaluate the effectiveness of hysteroscopic metroplasty on reproductive outcome in women with unexplained infertility with dysmorphic uteri.

## Materials and Methods

This retrospective study was conducted at Başkent University, Adana Dr. Turgut Noyan Practice and Research Hospital, Clinic of Obstetrics and Gynecology, Division of Reproductive Medicine and In Vitro Fertilization (IVF) Unit. Records of women who underwent hysteroscopic metroplasty for dysmorphic uterus with unexplained infertility from January 2013 to January 2016 were investigated. Mild and/or severe male infertility factor, diminished ovarian reserve, history of endometriosis and/or endometrioma, and tubal pathologies, including bilateral tubal obstruction or hydrosalpinx were excluded and eligible women with unexplained infertility were analyzed. All of the included women were assessed in two groups as primary and secondary infertility. Institutional review board approval This study was approved by Başkent University Institutional Review Board and Ethics Committee approval (KA: 17/151-A) and supported by Başkent University Research Fond was obtained for this retrospective cohort study.

### Pre-operative diagnosis

Infertile couples were admitted to Başkent University Division of Reproductive Medicine and IVF Unit. Medical history and ovarian reserve measured using the antral follicle count were evaluated, and hysterosalpingography (HSG) for the uterine cavity and tubal patency and sperm analysis were conducted. Unexplained infertility was defined as follows: unprotected intercourse for at least 1 year without conceiving with a normal ovarian reserve (i.e., antral follicle count over 4 in at least one functioning ovary with a regular menstrual cycle) and bilateral tubal patency observed on HSG and normal sperm analysis based on the World Health Organization 2010 criteria^(8)^. Dysmorphic uterus, formerly known as “T-shaped uterus,” was defined as a condensed lateral side uterine cavity observed using a properly performed HSG, in which the traction to the uterus was sufficient to expose the normal plain of the uterine cavity (Picture 1). The schematic view of the uterus revealed a T-shape, and the angle of the narrowed side wall was almost 90° (Picture 1). We were unable to perform 3D ultrasonography owing to the lack of this technology in our institution. There was no history of diethylstilbestrol (DES) exposure owing to the absence of DES use in Turkey. We think that the relationship between DES exposure and dysmorphic uterus remains speculative. Another issue associated with dysmorphic uterus class U1b, which is the infantile type, was that it could not be evaluated using HSG. This was a limitation of HSG. However, we did not experience any lateral wall perforation.

### Operation

Sedation with proper a dosage of propofol and midazolam was applied to the women. After vaginal scrubbing with a povidone-iodine solution, the office hysteroscope was inserted in to the uterine cavity using a non-touch technique. Office hysteroscopy was performed at the early follicular phase (days 6-14) using a 5 mm diameter continuous-flow hysteroscope with an oval profile and a 30° fore-oblique telescope and a 5-Fr operating channel (Office Continuous Flow Operative Hysteroscopy ‘size 5’; Karl Storz, Tuttlingen, Germany). Cavity distension was induced using an electronic irrigation and aspiration system (Endomat; Karl Storz, Tuttlingen, Germany) with NaCl 0.9%. A stable intrauterine pressure of ~40 mm Hg was obtained by setting the flow rate to 220-350 mm Hg; the negative pressure suction was set at 0.2 bar, and the pressure of irrigation was set at 100 mm Hg. After the evaluation of the uterine cavity, the lateral side walls were cut using a Bipolar Versapoint System (Gynecare Versapoint Bipolar Electrosurgery System, Ethicon, US) until the tubal orifice was visible from the isthmus. The video can be viewed at https://youtu.be/wVh9m0dxB2I. The operations were performed by four experienced surgeons. At the end of the operation, no anti-adhesive gel and/or intrauterine balloon was used. We did not record our operation duration; nevertheless, the mean duration of the operations was ~5 minutes.

### Post-operative approach

After the operation, the patients were discharged on the same day. The patients were prescribed 2 mg estradiol valerate oral estrogen support for 3 weeks (Cyclo-progynova tb, Bayer, Turkey). After 2 menstrual cycles, HSG was repeated for the evaluation of the newly formed uterine cavity (Picture 1). All patients were counseled to prevent them from conceiving until the second HSG. After evaluation of the new cavity using the second HSG, all patients were advised to try to conceive without assisted reproductive technology (ART) support for at least 1 year.

### Follow-up for the evaluation of reproductive outcomes

The reproductive outcomes were recorded using the delivery registry from our hospital database and via telephone interviews. Clinical pregnancy (CP) was defined as pregnancyiagnosed through ultrasonographic visualization of one or more gestational sacs or a positive blood human chorionic gonadotropin test result. The live birth rate (LBR) was noted as the delivery of at least one live born baby after 24 complete weeks of gestational age. The spontaneous abortion rate was defined as the spontaneous loss of a CP before 20 weeks of completed gestation. The delivery rate ending with live births at full term (37 weeks’ gestation) was also recorded.

### Statistical Analysis

The data are expressed as mean ± standard deviation. The baseline differences between the groups were analyzed using Student’s t-test. Pearson’s chi-square test and Fisher’s exact test were used to compare the ratios between the groups. Time to pregnancy after metroplasty was measured using Kaplan-Meier survival analysis. A p value of <0.05 was considered statistically significant. The data were analyzed using the SPSS for Windows (version 23.0; SPSS, Inc., Chicago, IL).

## Results

**Figure 1:**
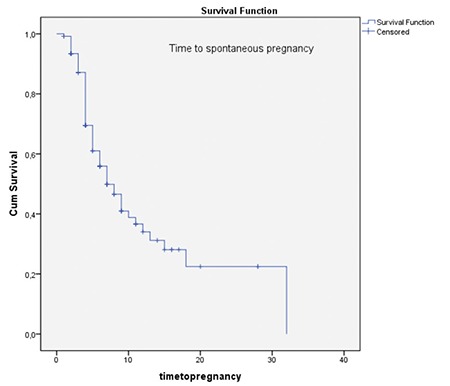
Time to spontaneous pregnancy

**Figure 2:**
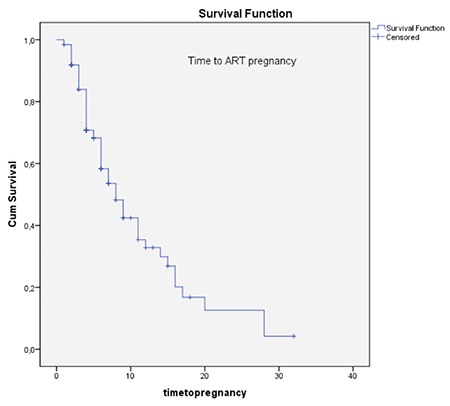
Time to assisted reproductive technology pregnancy

During the 3-year study period, 685 office hysteroscopies for dysmorphic uterus were performed to 272 women with unexplained infertility by our IVF department. The baseline characteristics of the patients are shown in Table 1. Of all the patients, 162 had primary infertility, and 110 had secondary infertility. The mean age and mean duration of infertility of the patients were similar. Forty-five women in the primary infertility group and 27 women in the secondary infertility group underwent an IVF/intracytoplasmic sperm injection (ICSI) cycle before metroplasty [27.8% (45/162) and 24.5% (27/110), respectively] (Table 1). In primary infertility group, 45 patients had IVF failures; 23 patients had a history of one cycle failure, 16 had two IVF/CSI failure cycles, and six patients had 3 IVF/ICSI failure cycles before metroplasty. In the secondary infertility group, 27 patients had a history of ART failures; 15 women had one IVF/ICSI failure, eight patients had two failure cycles, and four had three failure cycles. Seven of nine spontaneous pregnancies after metroplasty in the primary infertile women group had just one IVF failure and two patients had two failed IVF/ICSI cycles. In secondary infertile group, the number of spontaneous pregnancy after metroplasty in previous IVF failure cycles was six, four of which had one previous failure and two had 2 IVF/ICSI failures. The CPR and LBR after metroplasty are shown in Table 2. In the secondary infertility group, the miscarriage rate and ectopic pregnancy rate declined dramatically (p=0.01) (Table 2). Nine out of 45 patients (20%) in the primary infertility group and six out of 27 patients (22.2%) in the secondary infertility group with a history of IVF/ICSI failures successfully delivered subsequent to metroplasty through spontaneous pregnancies (Table 1). After metroplasty, the pregnancies of 44 patients in the primary infertility group and 24 patients in the secondary infertility group were successful with the first fresh IVF/ICSI cycle without waiting for spontaneous pregnancy. After the second HSG, the time to pregnancy was 5.8±4.7 months in the spontaneous pregnancy group and 6.7±5.7 months in the pregnancies with ART group (Figure 1, 2). The time to pregnancy in the primary and secondary infertile groups was also similar (Table 2).

## Discussion

Our study demonstrated that office hysteroscopic metroplasty of dysmorphic uterus might improve fertility, particularly in patients with unexplained infertility and with dysmorphic uterus, which was an ignored factor previously. Müllerian anomalies can impair obstetric outcomes because it can cause recurrent pregnancy loss (5-10%), as well as late miscarriage and preterm delivery (25%)^(9,10)^. These adverse obstetric outcomes may be subsequent to restricted expansion of an abnormally small endometrial cavity^(11)^. Increased contractility and decreased vascular perfusion of the fibrous uterine septum may also contribute^(12)^. We hypothesize that the enlargement of the uterine cavity using hysteroscopic metroplasty might restore obstetric outcomes by increasing the possibility for embryo implantation by proper endometrial vascular perfusion together with decreased uterine contractility inducing fragility in a dysmorphic uterus. This hypothesis might be supported by the majority of metroplasty outcomes of uterine septum surgery even in an arcuate uterus in ART cycles^(13,14)^. The higher prevalence of previous ectopic pregnancy in women with dysmorphic uterus was first reported by Fernandez et al.^(6)^. Our study also demonstrated that women with secondary infertility with dysmorphic uteri have higher ectopic pregnancy rates (15.5%). After correction of the uterine cavity, the ectopic pregnancy rate dropped to the expected population. This increased rate of ectopic pregnancy might be explained by the increased uterine contractility and decreased endometrial implantation in a dysmorphic uterus. Due to a lack of adequate and credible data, we did not apply any adhesion prevention strategies, such as intrauterine gel or balloon. We evaluated the newly-formed cavity configuration using a second HSG and hence did not observe any adhesions. We did not encounter any complications from hypotonic solution use such as electrolyte changes and hyponatremia because we applied saline during metroplasty procedures. Throughout the 3 years of the study, we did not encounter any complications of hysteroscopy such as uterine perforation or excessive bleeding after the operation. As expected, there would be no further risk of cervical incompetency owing to the thinner hysteroscope, thus ruling out the need for cervical dilatation. The major limitation of this retrospective study is the absence of the confirmation of a narrowed cavity using 3D transvaginal ultrasonography. Although our diagnostic criterion was based only on HSG, despite HSG being superior for the confirmation of dysmorphic uterus, 3D ultrasonography was feasible and appeared to be accurate as. If we had the opportunity to evaluate dysmorphic uterus using 3D ultrasonography, we would have calculated the volume of the endometrial cavity and provided a cut-off volume to compare with that of fertile women’s cavities. Although the importance of lateral wall thickness in dysmorphic uterus was included in the ESHRE/ESGE classification, the definitive ratio or thickness in the current literature was not mentioned. Furthermore, there is high intra/inter-observer variability in 3D ultrasonographic measurements of uterine wall thickness for uterine anomalies with conflicting data for their measurements owing to the inadequacy of the technique^(15)^. Although the ESHRE/ESGE consensus definitive diagnostic criteria were demonstrated using the schematic view for dysmorphic uterus, we speculate that as a diagnostic tool, the combination of HSG and 3D ultrasonography will achieve significantly better outcomes, which might be highlighted by future trials. Our department is an endoscopic surgery and ART clinic to which women who have a potentially higher prevalence of uterine anomalies are referred^(16)^. This is why the population ratio in this study seemed to be relatively higher. Furthermore, DES exposure was not documented in these women due to the absence of DES marketing in Turkey. In terms of obstetric outcomes, the risk associated with hysteroscopic metroplasty of dysmorphic uterus is the potential for placental adhesion owing to the possibility of electrosurgical injuries to the endometrium and myometrium. We had three different cases of placenta accreta that could be related to the intrauterine operation during the cesarean process. It is possible to see this type of potential adhesion even in the history of cesarean procedures conducted to date, hysteroscopic myomectomy, or any type of endomyometrial injury. The resultant outcomes at our department have been encouraging for fertility expectation in women with dysmorphic uterus. The ignored uterine-factor infertility must be considered in terms of dysmorphic uterus. The relationship between intrauterine adhesion and infertility seems to be similar to dysmorphic uterus related to lesser cavity volumes^(17)^. Further, the subsiding rate of ectopic pregnancy and miscarriage is also optimizing evidence for enlargement of dysmorphic cavities. Future randomized controlled trials are needed to support the effectiveness of the operation.

## Conclusion

Infertile women with dysmorphic uterus should be operated by hysteroscope to improve reproductive outcomes. Office hysteroscope is an alternative option in terms of non-invasive procedure.

## Figures and Tables

**Table 1:**
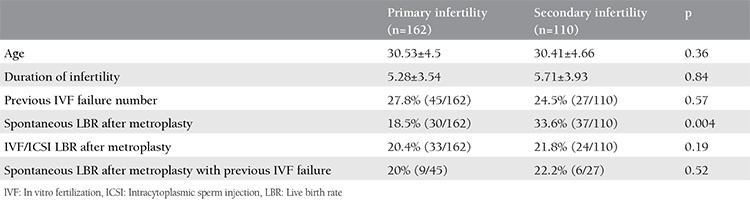
Basal characteristics of the study groups

**Table 2:**
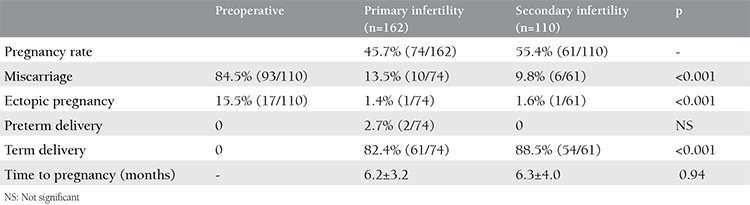
The reproductive outcomes of the secondary infertility group

**Picture 1:**
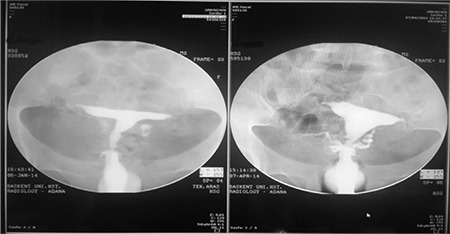
Preoperative and postoperative hysterosalpingography image *HSG: Hysterosalpingography*
